# The Effects of Planning and Handwriting Style on Quantity Measures in Secondary School Children’s Writing

**DOI:** 10.3389/fpsyg.2019.01143

**Published:** 2019-06-21

**Authors:** Gareth J. Williams, Rebecca F. Larkin, Emily Coyne-Umfreville, Toni C. Herbert

**Affiliations:** ^1^ Department of Psychology, Nottingham Trent University, Nottingham, United Kingdom; ^2^ School of Social Sciences, Birmingham City University, Birmingham, United Kingdom

**Keywords:** writing, spelling, transcription, translation, proposer

## Abstract

The study aimed to evaluate the proposer, translator, editor, and transcriber process model of writing in the context of secondary school children. Eighty-three children completed written texts under conditions that facilitated the proposer and placed resource demands on the transcriber. It was found that the number of words, lexical richness, and the number of sentences were affected by transcription resource demands, while the number of sentences was increased when the proposer was facilitated. There were also by-gender interactions that indicated male writers and female writers completed the tasks to different product levels. The discussion proposes that future developments of the model take into account a more direct interaction between the transcriber and translation level processes when considering this age group.

## Introduction

Writing is an important and complex skill that develops over childhood and into adulthood (Kellogg, 2008). It is a skill that requires sustained educational commitment over a number of years (Whitaker et al., 1994). Fitzgerald and Shanahan (2000) identified that in the early years of secondary school, children become more aware of writing to convey meaning to an audience. However, the mechanism by which secondary school writers produce text in this way is not clearly established.

Secondary school writers represent an opportunity to understand a point in development where writers are developing competencies in writing since early primary school education (Puranik and Lonigan, 2011). Writers in the early years of secondary school are close to automating their handwriting (Graham et al., 1998; Limpo et al., 2017). However, they are still developing further skills (Whitaker et al., 1994; Fitzgerald and Shanahan, 2000; Kellogg, 2008), particularly in relation to how they understand the audience relative to the writing they produce. Moreover, that they are still novices in the view presented by Bereiter and Scardamalia (1987), where the writer relies on long-term memory retrieval of information that is relevant to a prompt, termed knowledge telling. This contrasts with more sophisticated problem-solving strategies that expert writers use in addition to memory retrieval, knowledge transforming. Yet, there are other differences in this group of writers. In line with a theme that runs through primary and secondary education, female writers tend to outperform male writers in a range of writing outcomes (Berninger and Fuller, 1992; Knudson, 1995; c.f. Jones and Myhill, 2007; Williams and Larkin, 2013; Kim et al., 2015; Cordeiro et al., 2018; Lichtsteiner et al., 2018).

**Figure 1:**
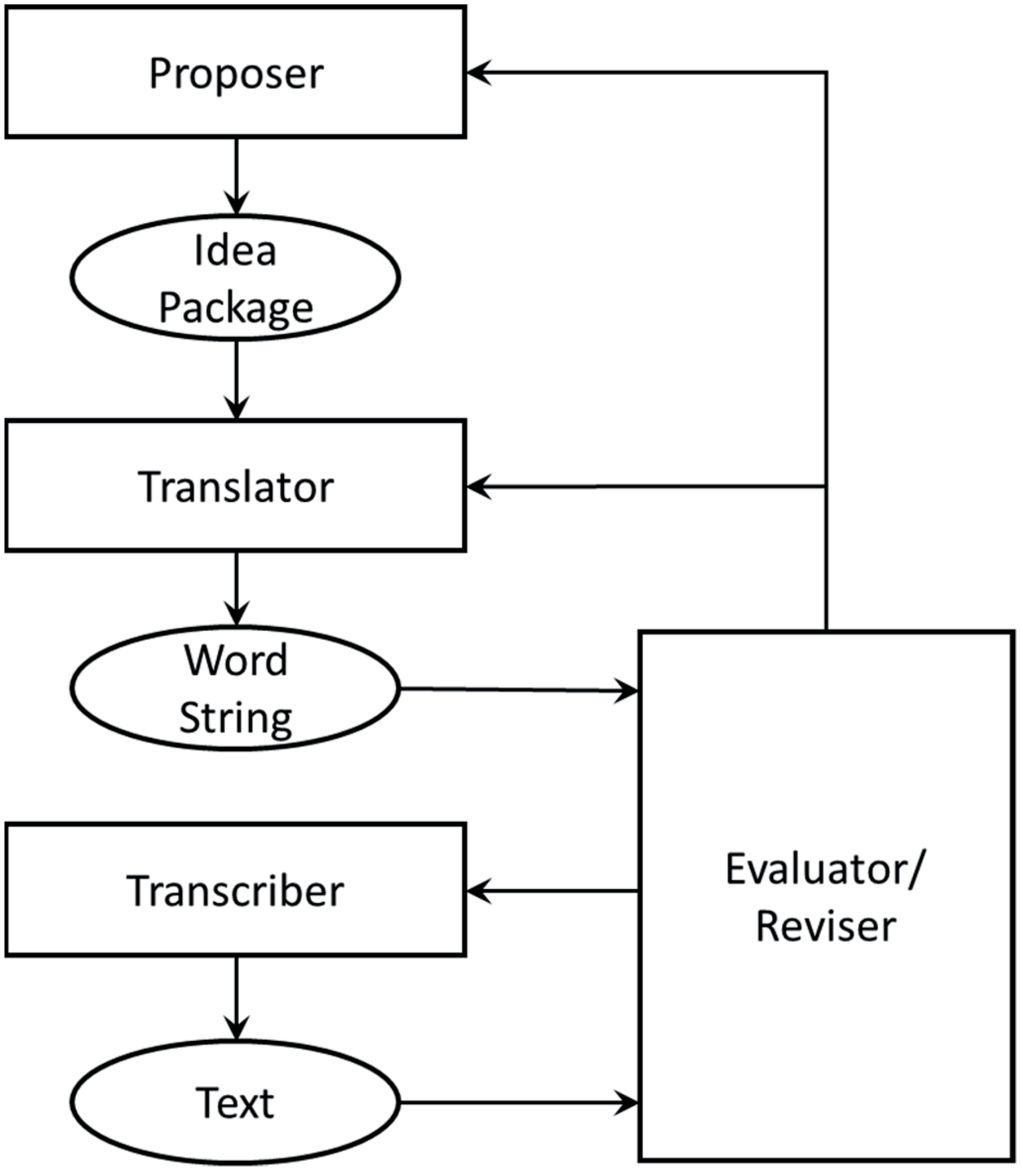
Chenoweth and Hayes (2003) model of writing (source Chenoweth and Hayes, 2003; Galbraith, 2009).

Chenoweth and Hayes (2003) put forward that writing is the outcome of processes that include a proposer, a translator, a process for revising and evaluating, and a transcriber (see Figure 1). Ideas are provided from the proposer and are translated into a word string for evaluation. If the word string meets the criteria for inclusion, it is then passed to the transcriber so that it is processed into text (see also Hayes and Flower, 1980; Hayes, 2011). In their 2003 study, they found support for the model by varying the task demands on the translation processes in adults. This paper reports experimental work that explores how changing the demands of transcription level processes and facilitating proposal level processes affects writing outcomes in secondary school children.

Transcription relates to processes involved in converting linguistic information from translation processes to writing output on the page or the screen (Abbott and Berninger, 1993; Chenoweth and Hayes, 2003; Limpo et al., 2017). Transcription includes spelling processes, which draw on language skills and the understanding of conventions used when representing language in writing (Caravolas et al., 2001), as well as the physical aspects of handwriting or keyboarding (Chenoweth and Hayes, 2003; Berninger and Winn, 2006).

Children typically handwrite in a classroom environment and for examinations. Although some work involves computers, children engage in handwriting throughout their school life (Christensen, 2009). In the United Kingdom, there is a requirement that children are able to produce handwriting neatly and fluently by the end of primary school (Department for Education, 2014). Although approaches vary by school, children tend to learn to print letters in isolation first (manuscript) and then join up their writing to produce cursive writing (Smith, 1987). This pattern is reported in a number of studies over time (Ames and Ilg, 1951; Karlsdottir, 1996) and is in comparison to countries, such as France, where cursive writing is taught exclusively from the start of school (Morin et al., 2012). However, teachers report many children begin to develop their own style, often reverting to a manuscript style, once they reach secondary school (Graham and Weintraub, 1996; Bara and Morin, 2013).

Transcription processes have, only recently, been shown to have an effect on writing outcomes. Transcription speed is related to children’s writing ability (Graham et al., 1997; Babayiğit and Stainthorp, 2010) and writing automatization increases over primary school, even though there remains considerable variation in speed of writing between children (Graham et al., 1998). Moreover, Limpo and Alves (2013), Alves and Limpo (2015), and Limpo et al. (2017) have shown that transcription processes contribute more to writing outcomes when children are first learning to write, and this contribution decreases as children progress through school. Even so, transcription processes continue to contribute to writing, albeit indirectly, when children are in early secondary school (Limpo et al., 2017). Moreover, Alves et al. (2016) demonstrated that handwriting interventions improved transcription skills, leading to improved handwritten text outcomes, significantly more so than keyboarding or spelling interventions. Transcription speed itself has been measured in several different ways. One is to use technology to measure the execution speed of the handwriting itself (e.g., Alves and Limpo, 2015; Grewal and Williams, 2018). Complementary approaches include using a specific task, such as letter copying speed (Graham et al., 1998) or writing a letter string from memory (Graham et al., 1997), for example, the letters “a” to “z” (Graham et al., 1997; Graham et al., 1998). It is also possible to vary the demands that transcription places on cognitive resources during writing.

A series of studies by Bourdin and Fayol (1994, 2000, 2002) demonstrated that cognitive resources are drawn upon during writing and, where transcription demands more of this capacity – or the need to translate presented information draws more resource (Bourdin and Fayol, 2002) – this then affects the overall output produced. Bourdin and Fayol (1994, 2000, 2002) found that, while writing output is more demanding than verbal output for seven-year old children, both outputs made similar demands on adults. Bourdin and Fayol (1994) went further and demonstrated that writing transcription demand could be increased by asking adult participants to write in a script that they had practiced less; in their study, this was cursive upper-case writing. In the more demanding condition, participants wrote significantly fewer words. Bourdin and Fayol (2002) demonstrated that changing the demands of the information presented to adult participants, whether they wrote with prompts that had semantically related words or unrelated pairs of words, also affected their ability to write text.

Although children were able to produce longer texts when making verbal reports (Bourdin and Fayol, 1994), Bourdin and Fayol (2000) found that a verbal report condition could be made more difficult, in line with a writing condition, if children were asked to complete a concurrent task. Tasks that were more like writing – drawing or monitoring for specific information – affected output, while repetitive tapping – not expected to interfere with production processes – did not affect verbal responses. Olive and Kellogg (2002) found that when participants were asked to either copy a text, pause and compose a text, or compose a text without pausing, children’s response times were significantly longer than adults to acoustic probes overall. Although adult writers were quickest at responding to probes when copying texts, response times were attenuated when they were asked, in a more demanding condition, to write in an unfamiliar script. Therefore, as with Bourdin and Fayol (1994), where the transcription process requires more resources it affects other processes; in Olive and Kellogg (2002) this was the ability to attend to another stimulus.

Alves et al. (2008), using an adapted method of the probe task used in Olive and Kellogg (2002), asked adult participants to identify which processes were taking place during writing and execution pauses. Following training, participants then explained what process of writing they were carrying out following each auditory probe in a typing task where they composed text to a picture-based prompt. Alves et al. (2008) found that during motor execution, writers often carried out translation processes at the same time. Planning or translating was more likely to occur when a participant had paused. In a study where participants were asked to handwrite to a prompt, Olive et al. (2009) found that increasing the task demands, by asking adult participants to write in an unfamiliar script, changed the explanations they gave to an auditory probe. When writing in a familiar script, participants often reported concurrent processes, such as translating while executing motor actions. However, when writing in an unfamiliar script, participants reported that processes took place serially instead. Taken together, these two studies demonstrate that transcription and translation often occur together and that as task demands increase, writing processes that would otherwise be able to run in parallel require more time as they become sequenced to when cognitive resources are available.

Planning is most associated with the proposer in the Chenoweth and Hayes (2003) model. This is in the context that writing requires the author to set a goal, structure their writing, and that this process is connected to the executive functions in writing (Hayes and Flower, 1980). Typically, children do not plan writing unless directed to do so (Berninger et al., 1994). Berninger et al. (1994) found that, in secondary school children, there was a writing outcome advantage for writers when they were asked to plan. Furthermore, in an intervention study, Limpo and Alves (2013) found that planning interventions were able to support young writers to develop better writing outputs. However, Limpo et al. (2014) found that planning could also be viewed from a developmental context; upper primary school children, where planning did not significantly predict writing outcomes, and lower secondary school children, where there was a significant association between planning and writing outcomes. Limpo et al. (2014) suggested that planning supports writers who are beginning to engage in knowledge transforming as opposed to younger writers who as still engaged primarily in knowledge telling.

In summary, Chenoweth and Hayes’ (2003) model makes the case that increasing demands on transcription level processes draws on cognitive resources that impacts other writing processes (Bourdin and Fayol, 1994; McCutchen, 2006). Typically, this would be translation processes, shown to be both active during transcription (Olive et al., 2009) and affected by demands for resources from other processes (Bourdin and Fayol, 2002; Chenoweth and Hayes, 2003; Olive et al., 2009).

The aim of the study was to investigate how facilitating the proposer and changing the demands made on the transcriber affected the profile of writing outcomes in secondary school children. It was predicted that changing demands at the transcription level (Bourdin and Fayol, 1994, 2000, 2002; McCutchen, 2006), by asking the participants to write in their non-preferred handwriting style, would result in changes at the word-level of production. Children who were writing in a less preferred style would produce fewer words and less diverse writing. Spelling, more closely associated with transcription level processes (Berninger and Winn, 2006), was predicted to also be affected by this condition. Moreover, following Berninger et al. (1994) and Limpo and Alves (2013), asking participants to plan would affect the number of ideas and sentences produced, as these are outcome measures associated with the proposer (Hayes and Flower, 1980). Moreover, researchers have indicated that male writers and female writers approach text production differently (Jones and Myhill, 2007). Therefore, it was predicted that there would be different patterns of responses in the outcome measures for these two groups.

## Materials and Methods

### Design

The within-participant independent variables were handwriting style (cursive, manuscript), preferred style (preferred, non-preferred), and planning (no plan, plan). The between-participant independent variable was gender (male, female). The dependent variables were the number of words, the lexical richness of the text, the number of ideas and sentences, and the proportion of spelling errors.

### Participants

The participants who took part in the study came from one secondary school in the East Midlands of the United Kingdom. In total, there were 127 participants, and they were removed from the analysis if they were missing responses to any of the tasks (*n* = 36) or did not plan when in the planning conditions (*n* = 3). Participants were also removed if they had no clear style of writing (*n* = 5). In total, there were 83 participants available for analysis (43 females and 40 males). In this sample, there was no significant difference in age between male participants and female participants (males *mean* = 13 years and 7 months, *SD* = 9.95 months; females *mean* = 13 years and 5 months, *SD* = 11.24; *t*(81) = 0.93, *p* = 0.36, *d′* = 0.20, overall age range from 11 years 11 months to 15 years and 11 months).

The participants were in mainstream school, and no exclusion criteria were applied. Fifteen participants reported that they also spoke a language other than English; however, all the children who took part were proficient in English. Data from the Department for Education indicated that the children made average progress in the school and had a slightly lower proportion of children classed as disadvantaged than the national average.

### Alphabet Task

The task was devised in line with Graham et al. (1997). The children were asked to write the alphabet from “a” to “z” in lowercase and to continue to do so for 30 s in their preferred writing style. The dependent variable was the number of correct letters written. However, there were almost no errors in the responses. Graham et al. (1997), with a similar method, reported an inter-rater Cronbach’s alpha of 0.98 in scoring errors.

### Letter Copy Task

The children were provided with a sheet of paper with manuscript letters in an array that were in a random order. They were asked to copy the letters directly below the letter. The number of letters copied in 2 min was used as the letter copy task dependent variable.

### Written Texts

Five different written text prompts were used, and the prompts were given the context of letters addressed to a fictional school newspaper. The first prompt was used as a familiarization task and to establish whether a child tended to write in a mostly cursive or a mostly manuscript style. The four other prompts were then used for the handwriting style and planning conditions. The prompts were chosen as they contained subjects that the participants were likely to be engaged with. These were “Do you think physical education should be compulsory in schools?”, “Do you think the school day should finish at 6pm?”, “Do you think the school summer holiday should be three weeks shorter?”, “Do you think cities should give more space to parks than houses and shops?”, and “Do you think governments should restrict the amount of sugar in foods?”. The written texts were framed by an, already completed, address and signature line. This was so that the participants would focus on writing the main body of the text. To address potential order effects, the order of prompts and whether a participant was asked to write in cursive or manuscript were counterbalanced.

Lexical richness was measured using Guiraud’s measure of lexical richness (cited in Vermeer, 2000). To calculate the number of ideas, Bourdin and Fayol’s (2002) method was followed. The inter-rater reliability for this measure was 0.82 (intra-class correlation), based on 21% of the written texts.

### Procedure

Data collection took place on a class-wide basis at the school. After an initial explanation to the class about the project, for each written text prompt, the participants were asked to read the prompt and the instructions provided. Participants answered the first prompt, the familiarization task, and they were asked to write in whichever of the styles was most comfortable to them. The next four prompts made up the experimental task. The first two written texts formed the no planning condition. The participants were not directed to, neither was there space provided, plan their response. For one of these two essays, the participants were asked to write in a cursive style and, in the other, a manuscript style. In the next two written texts, an additional page in the task booklet and time were provided to plan. Participants were directed to complete the plan first and then write their response to the prompt. The alphabet task and the letter copy task were completed in between the writing prompt tasks.

Throughout the tasks, teachers and members of the research team were present in the room to monitor how well the participants followed the instructions and to address any queries. During the no-plan conditions, children showed no planning behaviors and no notes indicating planning were found in the participants’ texts. Moreover, there were no indications that children systematically revised their texts before the end of the planning period.

For each writing prompt, participants were provided with 5 min to write their answer. In the conditions with planning, participants were provided with 2 min to plan their written texts. It was typical for participants to finish planning their written texts within the time provided. However, they were also instructed not to begin their writing even if they had completed planning. In total, the tasks took around 50 min to complete.

This study was carried out in accordance with the ethical guidelines of the British Psychological Society. The protocol was approved by the College of Business, Law and Social Sciences Ethics Committee at Nottingham Trent University. The school provided informed written consent for the children to take part. Parents had informed written consent that allowed them to withdraw their child from participation prior to the beginning of data collection or their child’s data for a period following the completion of data collection. The children were provided with an opportunity to choose to take part and were free to withdraw their participation during the study.

## Results

### Comparisons Between Manuscript Writer and Cursive Writer Characteristics

This was based on the first written text that the children wrote. Their writing was judged as either mostly manuscript (male *n* = 24; female *n* = 30) or mostly cursive (male *n* = 16; female *n* = 13). In terms of writing speed measures, writers who wrote in a manuscript style (*mean* = 20.48, *SD* = 5.44) were not significantly faster than cursive writers (*mean* = 19.69, *SD* = 5.96) when measured using the alphabet task *t*(81) = 0.61, *p* = 0.79, *d′* = 0.14. Moreover, neither the manuscript writers (*mean* = 125.31, *SD* = 37.29, *n* = 54) nor the cursive writers (*mean* = 128.03, *SD* = 37.76, *n* = 29) copied significantly more characters *t*(81) = −0.32, *p* = 0.75, *d′* = 0.07. These findings are consistent with a much earlier study, Gates and Brown (1929), although more recently Morin et al. (2012) found cursive writers to be slower in composition. Moreover, there was no significant association between gender and whether a participant wrote in a cursive or manuscript handwriting style, *χ*^2^ = 0.87, *p* = 0.35.

### Changes From the First to the Last Written Text by Gender Difference

To examine whether writers produced a consistent amount of output over the five written texts, the familiarization task and all four experimental tasks, a 5 Written text (first to last written text) × 2 Writing Style (cursive, manuscript) × 2 Gender (Male, Female) ANOVA was conducted with each of the dependent variables: the number of words, lexical richness, number of ideas, number of sentences, and the proportion of spelling errors.

The findings, for the number of words, indicated that there was no significant main effect of written text, or there were no significant interactions. There was, however, a main effect of gender, *F*(1, 81) = 6.25, *MSE* = 1639.78, *p* = 0.01, $\eta_{p}^{2}$ = 0.11. A pairwise comparison (*α* = 0.009) indicated that, across the five written texts, female writers (*mean* = 65.70, *SD* = 22.95) wrote significantly more words than male writers (*mean* = 55.76, *SD* = 23.45). There was also a significant main effect of gender when the proportion of spelling errors were analyzed, *F*(1, 81) = 7.34, *MSE* = 0, *p* = 0.01, $\eta_{p}^{2}$ = 0.016. Male writers (*mean* = 0.04, *SD* = 0.03) had a significantly higher proportion of spelling errors than female writers (*mean* = 0.03, *SD* = 0.03) but no other main effects or interactions were found. For the number of ideas, there was neither a significant main effect for gender nor for writing style. There was a main effect for written text, *F*(4, 316) = 3.12, *MSE* = 1.76, *p* = 0.02, $\eta_{p}^{2}$ = 0.04. However, this result did not indicate a trend for fewer ideas over the texts as the pairwise comparison analyses indicated that text four (*mean* = 4.07, *SD* = 1.70) had a significantly higher number of ideas compared with text one (*mean* = 4.75, *SD* = 2.06). Finally, for the number of sentences, there was a main effect of gender, *F*(1, 81) = 7.24, *MSE* = 7.8, *p* = 0.01, $\eta_{p}^{2}$ = 0.11, where female writers wrote significantly more sentences (*mean* = 3.42, *SD* = 1.77) than male writers (*mean* = 2.68, *SD* = 1.50). For the number of sentences, there was also a main effect of written text, *F*(4, 324) = 2.72, *MSE* = 1.4, *p* = 0.03, $\eta_{p}^{2}$ = 0.03. However, the pattern did not indicate that later written texts had fewer sentences as Bonferroni corrected pairwise comparisons (*α* = 0.009) did not show a significant difference between conditions. For lexical richness as a dependent variable, there were no significant main effects or interaction over the five written texts.

### The Effect of Demand Changes in Writing

To investigate how changes to the demands of writing affected different writing outcomes, a series of mixed ANOVAs were conducted. For each dependent measure, a 2 handwriting style (cursive, manuscript) × 2 planning (no plan, plan) × 2 preferred style (preferred, non-preferred) × 2 gender (male, female) ANOVA was conducted. Each of the dependent measures, number of words, lexical diversity, number of sentences, and the proportion of spelling errors were analyzed in turn. Bonferroni corrected pairwise comparisons were conducted where significant differences and interactions were found.

#### Number of Words

**Figure 2:**
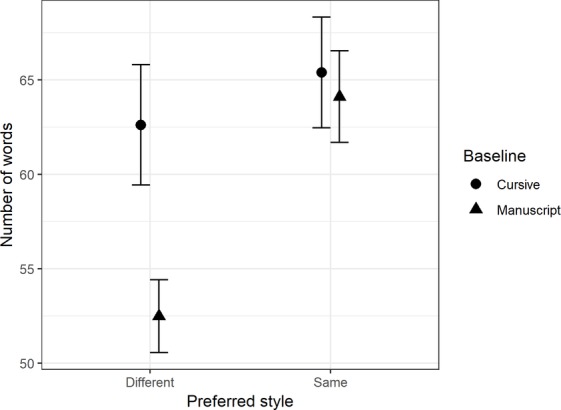
Comparison of means for the number of words, with 95% CI, between baseline handwriting style and preferred handwriting style.

Children wrote significantly more words in their preferred style than their non-preferred style, *F*(1,79) = 16.1, *MSE* = 245.94, *p* = 0.001, $\eta_{p}^{2}$ = 0.17. There was also a significant interaction between the handwriting style condition and whether a child preferred to write in a cursive or manuscript style, *F*(1,79) = 6.4, *p* = 0.01, $\eta_{p}^{2}$ = 0.08. The interaction (see Figure 2) demonstrated that, although there was no significant difference between the number of words written by cursive (*mean* = 65.40, *SD* = 22.36) and manuscript writers (*mean* = 64.11, *SD* = 25.16) when using their preferred style, manuscript writers produced significantly fewer words (*mean* = 52.49, *SD* = 20.10) when asked to write in their non-preferred style than cursive writers (*mean* = 65.62, *SD* = 24.26). There was also a significant main effect for gender, *F*(1,79) = 7.65, *MSE* = 1369.59, *p* < 0.01, $\eta_{p}^{2}$ = 0.09. Female writers (*mean* = 65.49, *SD* = 22.81) wrote significantly more words than male writers (*mean* = 56.71, *SD* = 23.04).

#### Lexical Richness

**Figure 3:**
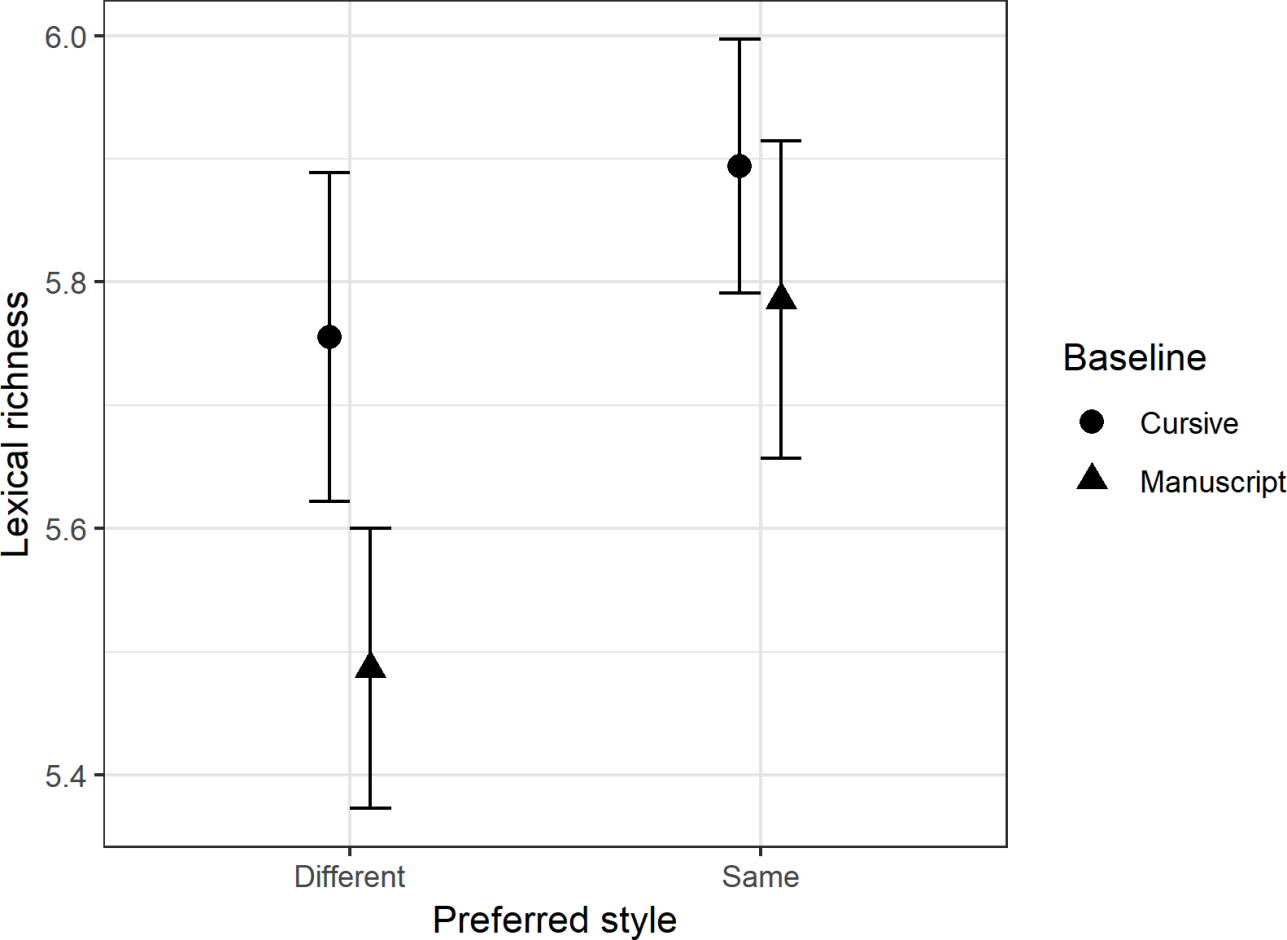
Comparison of lexical richness means, with 95% CI, between gender and preferred handwriting style.

There was a significant main effect for preferred style in lexical richness, *F*(1,79) = 10.5, *MSE* = 0.37, *p* = 0.01, $\eta_{p}^{2}$ = 0.12. Children had a significantly higher lexical richness in their preferred style (*mean* = 5.82, *SD* = 1.17) compared to their non-preferred style (*mean* = 5.58, *SD* = 1.13). There was also a significant interaction between gender and style preference, *F*(1,79) = 4.38, *p* = 0.04, $\eta_{p}^{2}$ = 0.05. Males were significantly less diverse when writing in their non-preferred style (*mean* = 5.41, *SD* = 1.30) compared to their preferred style (*mean* = 5.82, *SD* = 1.4), whereas females had a non-significant difference in lexical richness when writing in their preferred handwriting style (*mean* = 5.82, *SD* = 0.92) compared with non-preferred handwriting style (*mean* = 5.74, *SD* = 0.92). The results of the interaction are summarized in Figure 3.

#### Number of Ideas

**Figure 4:**
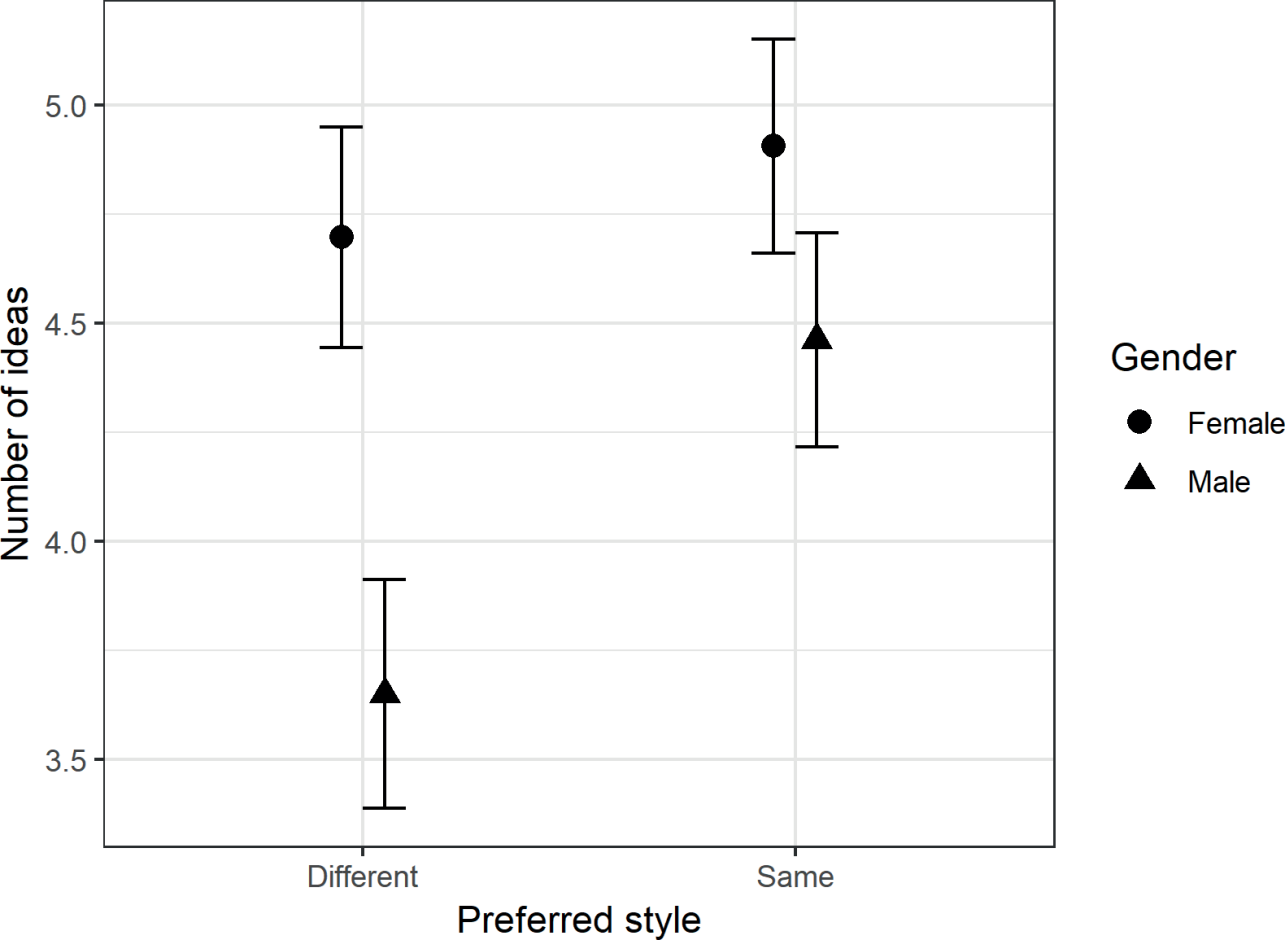
Comparison of means for the number of ideas, with 95% CI, between gender and preferred handwriting style.

There was a significant main effect for preferred style, *F*(1, 79) = 8.69, *MSE* = 1.88, *p* < 0.005, $\eta_{p}^{2}$ = 0.10, children wrote down significantly more ideas in their preferred style (*mean* = 4.69, *SD* = 1.85) than in their non-preferred style (*mean* = 4.19, *SD* = 1.92). Moreover, there was a main effect for initial handwriting style, *F*(1, 79) = 4.29, *MSE* = 7.95, *p* < 0.005, $\eta_{p}^{2}$ = 0.11. Cursive writers (*mean* = 5.03, *SD* = 2.13) wrote down significantly more ideas than manuscript writers (*mean* = 4.13, *SD* = 1.69). There was also a main effect for gender, *F*(1, 79) = 6.55, *MSE* = 7.95, *p* = 0.01, $\eta_{p}^{2}$ = 0.08, female writers (*mean* = 4.80, *SD* = 1.87) wrote significantly more ideas than male writers (*mean* = 4.06, *SD* = 1.86). Moreover, there was a significant interaction of preferred style by gender, *F*(1, 79) = 4.29, *p* = 0.04, $\eta_{p}^{2}$ = 0.05. Pairwise comparisons indicated that there was no significant difference for female writers whether they wrote in a preferred (*mean* = 4.60, *SD* = 1.78) or non-preferred style (*mean* = 4.40, *SD* = 1.85). However, there was a significant difference for male writers where they wrote significantly fewer ideas, in their non-preferred style (*mean* = 4.43, *SD* = 1.85) compared with their preferred style (*mean* = 3.70, *SD* = 1.77). The results of the interaction are summarized in Figure 4.

#### Number of Sentences

There was a significant main effect for preferred style, *F*(1,79) = 10.7, *MSE* = 1.57, *p* < 0.001, $\eta_{p}^{2}$ = 0.12, and children wrote significantly more sentences in their preferred style (*mean* = 3.24, *SD* = 1.90) than when asked to write in their non-preferred style (*mean* = 2.78, *SD* = 1.47). There was also a main effect of planning, *F*(1,79) = 4.04, *MSE* = 1.32, *p* = 0.048, $\eta_{p}^{2}$ = 0.05, children wrote more sentences after planning (*mean* = 3.15, *SD* = 1.79) compared with when they did not plan (*mean* = 2.87, *SD* = 1.62). Moreover, there was also a main effect of gender, *F*(1,79) = 10.58, *MSE* = 6.92, *p* = 0.01, $\eta_{p}^{2}$ = 0.12, female writers (*mean* = 3.40, *SD* = 1.80) wrote significantly more sentences than male writers (*mean* = 2.59, *SD* = 1.50). There was a significant planning by gender interaction, *F*(1,79) = 5.59, *p* = 0.02, $\eta_{p}^{2}$ = 0.07, in that there was no significant difference between male (*mean* = 2.64, *SD* = 1.69) and female (*mean* = 3.08, *SD* = 1.53) writers in the no planning condition but in the planning condition female writers (*mean* = 3.71, *SD* = 2.00) produced significantly more sentences than male writers (*mean* = 2.55, *SD* = 1.29).

**Figure 5:**
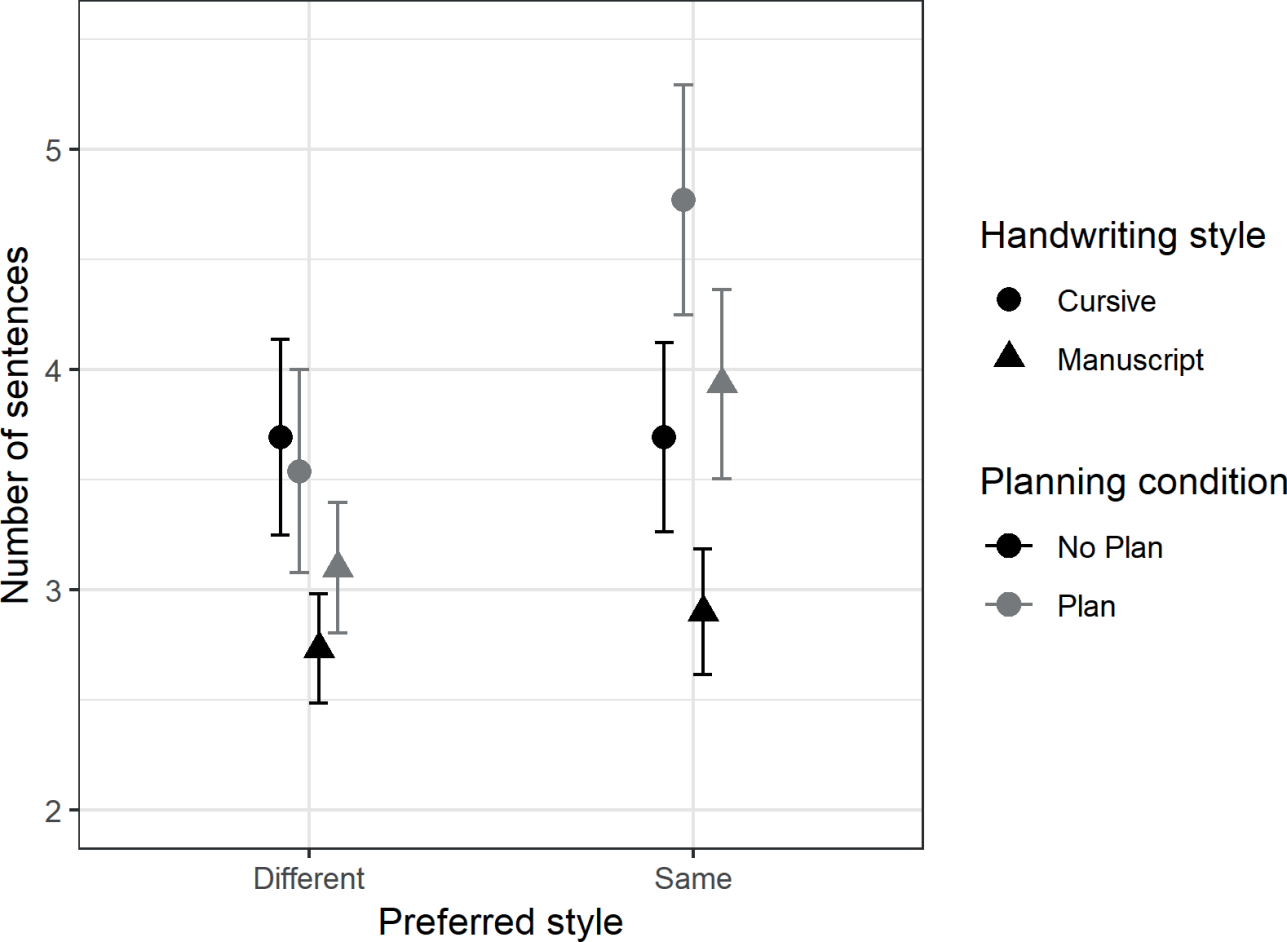
Comparison of means for the number of sentences, with 95% CI for female writers, between baseline handwriting style, planning, and preferred style.

**Figure 6:**
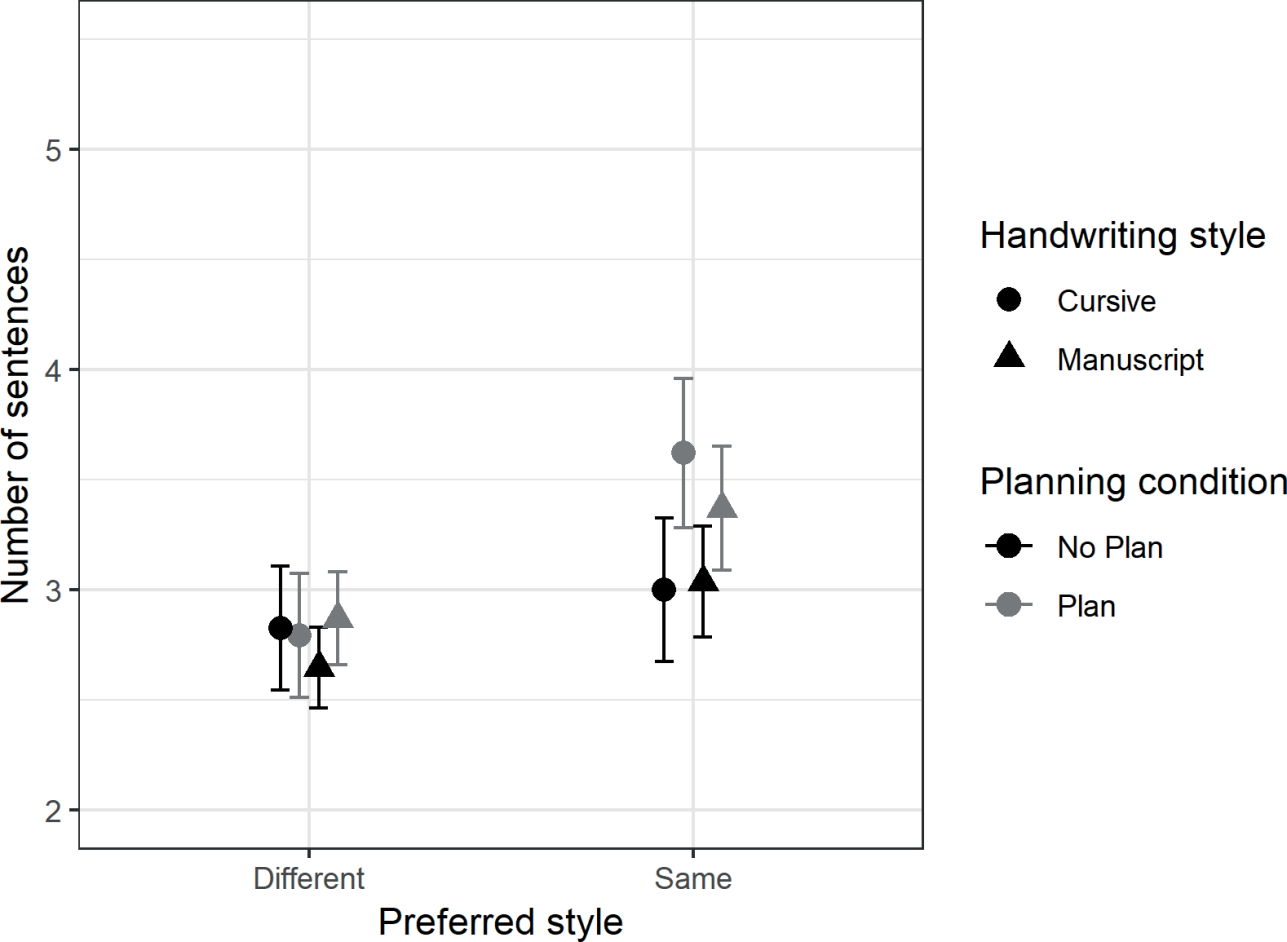
Comparison of means for the number of sentences, with 95% CI, for male writers, between baseline handwriting style, planning, and preferred style.

Finally, there was a significant three-way interaction between planning, preferred style, and gender, *F*(1,79) = 7.47, *p* = 0.01, $\eta_{p}^{2}$ = 0.09. Although female writers produced significantly more sentences when planning (*mean* = 4.19, *SD* = 2.29) compared to not planning (*mean* = 3.14, *SD* = 1.58) in their preferred writing style, there was no significant difference in the number of sentences they wrote in neither the planning (*mean* = 3.23, *SD* = 1.63) nor the non-planning (*mean* = 3.02, *SD* = 1.49) conditions when writing in their non-preferred style (summarized in Figure 5). In comparison, male writers (Figure 6) wrote a similar number of sentences in all four conditions (mean range from 2.38 to 2.90, SD range from 1.23 to 2.04).

#### Proportion of Spelling Errors

There was a main effect of gender, *F*(1,79) = 9.08, *MSE* = 0, *p <* 0.01, $\eta_{p}^{2}$ = 0.1, and male writers (*mean* = 0.04, *SD* = 0.03) had a higher proportion of spelling errors than female writers (*mean* = 0.03, *SD* = 0.03). There were no other significant main effects or interactions.

### Using Writing Speed or Letter Copying Speed as Covariates

For each of these analyses, the mixed ANOVAs were re-run separately with either the alphabet task or the letter copy task as a covariate. This was to examine whether the significant finding patterns would remain if each speed tasks were added to the models. The two measure had a non-significant correlation, *r* = 0.12, *p* = 0.28. This finding is in contrast to Graham et al. (1997) who reported a weak, significant, correlation between two measures of transcription speed, a paragraph copy task and a 15 s alphabet writing task, in their sample of 300 secondary school children. However, the researchers also found a developmental trajectory in that their primary school sample had a strong correlation between the two measures. It is likely that as children develop their writing skills, writing speed, and copying speed begin to measure different aspects of writing fluency.

#### Number of Words

The significant main effect for gender remained after the alphabet task was added as a covariate, and the number of words was the dependent variable. The significant pattern also remained for the main effect for preferred style and the interaction between preferred style and the style that children initially wrote. The pattern of significant effects and interactions remained the same with the letter copy task added as a covariate.

#### Lexical Richness

Adding the alphabet task as a covariate, where lexical richness was the dependent variable, resulted in the main effect for preferred handwriting style becoming non-significant, *F*(1, 78) = 0.389, *p* = 0.54, $\eta_{p}^{2}$ = 0, and the significant interaction between gender and preferred handwriting style also became non-significant, *F*(1, 78) = 3.81, *p* = 0.05, $\eta_{p}^{2}$ = 0.05. When the letter copy task was added instead, the main effect for preferred style also became non-significant, *F*(1, 78) = 0.42, *p* = 0.70, $\eta_{p}^{2}$ = 0.0, but the gender by style preference interaction remained significant.

#### Number of Ideas

For the alphabet task added as a covariate and the number ideas as the measurement, the main effect for preferred handwriting style became non-significant, *F*(1, 78) = 1.09, *p* = 0.3, $\eta_{p}^{2}$ = 0.01. When the letter copy task was added as a covariate, the main effect for preferred handwriting style also became non-significant, *F*(1, 78) = 1.46, *p* = 0.23, $\eta_{p}^{2}$ = 0.02. For either covariate measure, the preferred style by gender interaction remained significant as did the main effect for gender and the main effect for initial handwriting style.

#### Number of Sentences

When the alphabet task was added as a covariate to the analysis, where the number of sentences was the dependent variable, the main effect of planning remained significant, as did the main effect for gender, and also the significant gender by planning interaction. The interaction between planning, preferred style, and gender remained. However, the main effect for preferred style became non-significant, *F*(1, 78) = 0.40, *p* = 0.53, $\eta_{p}^{2}$ = 0.0. When the letter copy task was added, the main effect for planning was no longer significant, *F*(1, 78) = 1.32, *p* = 0.25, $\eta_{p}^{2}$ = 0.02. However, the main effect for gender and the planning by gender interaction remained significant, as did the three-way interaction between planning, preferred style, and gender.

#### Proportion of Spelling Errors

For the proportion of spelling errors, the main effect for gender remained significant when either the letter copy task or the alphabet task were added as covariates.

## Discussion

The study set out to investigate if writing outcomes that related to the proposer and the transcriber processes in writing (Hayes and Flower, 1980; Chenoweth and Hayes, 2003) were affected by changes to transcription resource demand (Bourdin and Fayol, 1994; McCutchen, 2006) and planning (Berninger et al., 1994; Limpo and Alves, 2013) in secondary school children.

In line with the predictions, it was found that changing the demand characteristics of handwriting affected the number of words written and the lexical richness of writing. However, it also affected the number of ideas in written text. Children wrote more words, had higher lexical richness, and wrote down more ideas in their preferred style compared to their non-preferred style of handwriting. Moreover, manuscript writers wrote significantly fewer words in their non-preferred (cursive) style of writing compared to when children who preferred cursive writing were asked to write in their non-preferred style. Furthermore, contrary to the predicted result, the proportion of spelling errors remained constant throughout the conditions.

In line with the predictions for planning. Asking children to plan their written responses affected the number of sentences a child wrote, but not the number of words, the lexical richness, nor the proportion of spelling errors. These children, overall, wrote more sentences after planning than in the no planning condition. However, planning did not significantly affect the number of ideas in written texts.

These findings were more complicated by the pattern of results found when comparing male writers and female writers. In this study, female writers wrote significantly more words, more sentences, and had a significantly lower proportion of spelling errors. When writing in their non-preferred handwriting style, female writers also wrote with higher lexical richness and wrote down more ideas than male writers. Female writers also wrote more sentences in their preferred handwriting style after they had planned their text.

When writing speed – using either an alphabet task or a letter copy task – were added to the analysis, this changed the pattern of results for some measures but not others. The pattern of significant results remained the same for the number of words and the proportion of spelling errors. However, for lexical richness, the number of ideas, and the number of sentences, the pattern of significant results changed as either writing speed measure was added to the analysis.

That changing the resource demands of transcription level processes primarily affected the outcome measures associated with the translator is consistent with the experiments conducted by Bourdin and Fayol (1994, 2000, 2002). They found that varying the handwriting task demands for adults and comparing verbal with written delivery in young children changed the amount of production. The findings in this study extend this to secondary school children. Therefore, overall, the findings are in line with the view that there is a limited cognitive resource that processes draw upon when engaged in writing (Hayes and Flower, 1980; Bourdin and Fayol, 1994, 2000, 2002; Olive and Kellogg, 2002; Chenoweth and Hayes, 2003). Moreover, the analysis of covariance results is in line with an account where writing fluency plays a role in word-level output (Graham et al., 2007).

Comparing planning conditions to no planning conditions indicated that children wrote more sentences when asked to plan. Placing sentence punctuation represents a circumstance where a writer imposes a structure on their text. However, children did not provide significantly more ideas when asked to plan. Overall these findings offer partial support for the role of planning in writing output (Berninger et al., 1994). As noted by Limpo et al. (2014), planning benefit writers who are engaged in knowledge transforming and it is possible that the children in this study are on the boundary between knowledge telling and knowledge transforming (Fitzgerald and Shanahan, 2000). Moreover, different forms of planning are likely to produce different outcomes (e.g., Limpo and Alves, 2013). However, the findings from this study indicate that planning per se might not always produce higher writing outcomes.

### Gender Differences in Writing

Previous research has found gender differences in writing where male writers often produce less writing output (Kim et al., 2015; Williams and Larkin, 2013; Cordeiro et al., 2018; Lichtsteiner et al., 2018). Therefore, gender differences were addressed in the models of analysis in this study. The results indicated that there tended to be a writing style preference by gender interaction. Male writers had texts with lower lexical richness when writing in their non-preferred style compared to their preferred style, and a similar pattern was found when the number of ideas was measured. Moreover, whereas planning was not affected overall by changes to writing style, there was an interaction with gender. Female writers wrote the most sentences, significantly, when they planned and they also used their preferred style of handwriting. However, for male writers, the number of sentences remained similar for each condition. It is unclear why there would be a gender difference. One possibility is that male writers and female writers approach the task in different ways (Jones and Myhill, 2007), and that this has been captured in this study.

### Implications for Writing Models

Children’s writing skills at secondary school level is an under-researched field. The model put forward by Chenoweth and Hayes (2003) described a feed-forward process between proposer, translator, reviser, and a transcriber. Where there is then a feedback loop, it is mediated by the reviser and this indirectly links the transcriber with the translator and the proposer. One of the model’s assumptions is that the processes are related to skilled adult writers. Therefore, it has been less clear how the models of writing relate to writers who are developing their skills. Overall, the findings provide support for the Chenoweth and Hayes (2003) model for children who are at this age. Changes to the resource demands at the transcription level, through writing in a non-preferred handwriting style, affected translator related outcomes. Where, as transcription takes more resources, this leads to a reduction in the resources available to produce word strings from the proposer, as was described by the participants in Olive et al. (2009). Therefore, the findings of this study indicate that there is an interactive relationship between transcription and translation. This would be consistent with the findings of the structural equation models reported by Limpo et al. (2017).

Typically, writing models place spelling at the transcription level (Berninger and Winn, 2006). However, the proportion of spelling errors was not affected by the direction to write in a non-preferred style of handwriting – another transcription process – and this suggests that spelling is processed elsewhere in the writing process in writers that are of secondary school age. One likely location is through an interaction between the translator, where ideas are translated into language information, and the reviser. This interaction is so that the reviser can trap and correct errors. It can then elicit alternative candidate words from the translator before this information reaches the transcriber.

It is notable that the percentage of errors in this study is low, suggesting that spelling errors are often revised before words are transcribed. Spelling is a skill that is composed of phonological and orthographic processes (Caravolas et al., 2001), and links to working memory (Ormrod and Cochran, 1988), reading fluency (Savage et al., 2008), and reading accuracy (Van Orden et al., 1988). It is possible that some translation processes, as well as transcription processes, play a role in accurate spelling during writing.

### Limitations

Changing the resource demand characteristics in handwriting did not create equal difficulty for children who wrote in a manuscript handwriting style compared with children who wrote in a cursive style. Although the two groups of children wrote as quickly as each other, as measured by both the alphabet task and the letter copy task, it was the children who wrote in a manuscript style who were more affected by being asked to write in a non-preferred style, as measured by the number of words they wrote. Manuscript writing tends to precede cursive writing as children develop their handwriting style (Ames and Ilg, 1951; Karlsdottir, 1996). It is likely that cursive writers were able to more easily switch to a manuscript style, while the transition to cursive writing required more effort for writers who tended to use a manuscript style. This points to an account where writers in a cursive style have developed proficiency in both manuscript and cursive styles. For secondary school writers who used a manuscript style, they had developed their handwriting to a sufficient level to be as fast as children who wrote in a cursive style, but it appears they had done so at the consequence of not developing equal proficiency in cursive writing.

The study did not directly assess children’s working memory (McCutchen, 2006), and so provides an incomplete picture of the constraints that writers are operating under. It is possible that, alongside assessing writing speed, working memory could offer a further covariate to account for variations in writing outcomes. Moreover, as writing is a complex task of interrelated processes (Berninger and Winn, 2006), the measures of writing outcomes themselves are likely to overlap with each other.

Moreover, although the children were in a mainstream school, it is also possible that some children who wrote texts required specialist literacy support and that this affected the overall pattern of results. Previous studies have indicated that children with literacy difficulties are affected at the translation or transcription levels. Williams and Larkin (2013) found that, although children with reading difficulties produced more spelling errors, they wrote texts that had a similar profile to typical children. Berninger et al. (2008) studied young writers who had dyslexia. They found these children had more spelling errors and poorer writing than would be expected for their age. Moreover, children with developmental language difficulties tend to write fewer words and produce lower quality texts compared with typically developing children (Williams et al., 2013). Finally, given the fine-motor coordination requirements of fluent writing, children with developmental coordination disorder have tended to report difficulties with writing that impact on their educational experiences (Dunford et al., 2005). Future studies that focus on children who require specialist literacy support would help to develop this area in more detail.

It is also possible that motivational aspects might affect writing quantity. Assessing motivation offers an opportunity to explore another aspect of the patterns in writing outcome measurements. Previous research has found that motivation often plays a role in the performance of literate activities (Bruning and Horn, 2000).

### Future Research

The study reported here investigated planning and transcription with secondary school children. It is possible to develop this approach further, to encompass a broader range of processes outlined in Chenoweth and Hayes (2003), by adapting the task for computer. In this way, for example, it would be possible to suppress the screen display (e.g., Chenoweth and Hayes, 2003) so that this affects the ability of writers to revise their written output. Moreover, it would be possible to compare the written production in handwriting with keyboarded written production. Finally, it is possible that a more detailed analysis of the writing bursts and pauses (Kaufer et al., 1986; Olive et al., 2009; Grewal and Williams, 2018) associated with writing output might provide a further view on transcription level processes and how they are connected to translation and proposal processes, in the context of the Chenoweth and Hayes (2003) model of writing.

## Conclusion

In conclusion, the findings indicated that changing transcription level resource demands affects some translation level processes. Specifically, it reduced the number of words produced and the lexical richness of written texts. Planning increased the number of sentences but not the number of ideas. Spelling error levels remained consistent over the different conditions. However, the pattern of results differed for female writers compared with male writers. Taken together, the findings support previous studies in transcription and planning, while also making the case for a more direct connection between translation and transcription in secondary school children.

## Ethics Statement

This study was carried out in accordance with the ethical guidelines of the British Psychological Society. The protocol was approved by the College of Business, Law and Social Sciences Ethics Committee at Nottingham Trent University. The school provided informed written consent for the children to take part. Parents had informed written consent that allowed them to withdraw their child from participation prior to the beginning of data collection or their child’s data for a period following the completion of data collection. The children were provided with an opportunity to choose to take part and were free to withdraw their participation during the study.

## Author Contributions

GW designed the study, conducted data collection, statistical analysis, wrote the original manuscript, and contributed to manuscript draft revisions. RL was involved in designing the study and contributed to manuscript draft revisions. EC-U was involved in designing the study and contributed to manuscript draft revisions. TH assisted in designing the materials, supported data collection, and contributed to manuscript draft revisions.

### Conflict of Interest Statement

The authors declare that the research was conducted in the absence of any commercial or financial relationships that could be construed as a potential conflict of interest.
